# LncRNA AK001796 promotes cell proliferation via acting as a ceRNA of miR-150 in hepatocellular carcinoma

**DOI:** 10.1590/1678-4685-GMB-2022-0277

**Published:** 2023-06-02

**Authors:** Rui Xu, Haitao He, Yue Wang, Qi Peng, Ke Mei, Yan Liu, Qing Yang

**Affiliations:** 1 Jilin University, College of Basic Medical Sciences, Department of Pathogenobiology, Changchun, Jilin Province, China.; 2 Jilin University, College of Basic Medical Sciences, Department of Cell Biology, Changchun, Jilin Province, China.

**Keywords:** AK001796, ceRNA, hepatocellular carcinoma, miR-150, proliferation

## Abstract

Long non-coding RNA AK001796 was initially identified altered in lung cancer. Recent research showed it could participate in the prognosis of hepatocellular carcinoma (HCC). However, the general biological role of AK001796 and its underlying mechanisms in HCC remain unclear. Here we demonstrated that the expression level of AK001796 in HCC tissues and cell lines was up-regulated. Silencing AK001796 suppressed the proliferation ability of HCC cells. Through dual luciferase reporter assays and loss/gain of functions studies, we identified that AK001796 could bind to miR-150, a star microRNA, promoting HCC proliferation. Furthermore, it was reported that growth factor receptor binding protein 2-associated binder 1 (GAB1) is a target gene of miR-150. Owing to AK001796 being a decoy for miR-150 and binding the same putative sites of miR-150 as GAB1, we presented that inhibition of miR-150 in AK001796 silencing cells reversed the reduction in GAB1. Subsequently, our findings demonstrated that silencing AK001796 can impair phospho-ERK1/2 and phospho-AKT. In conclusion, our investigation revealed that AK001796 promoted proliferation by enhancing phospho-ERK1/2 and phospho-AKT through AK001796/miR-150/GAB1 axis in HCC. These results provided further evidence for the critical roles of AK001796 accumulating HCC and suggested that AK001796 might act as an HCC biomarker in clinical treatment.

## Introduction

Hepatocellular carcinoma (HCC) is the fifth most commonly diagnosed cancer and the fourth leading cause of cancer mortality around the world. It mainly occurred in eastern Asia, especially in China (Bray et al., 2018). Through providing HBV and HCV vaccination in infants, the mortality-rate of liver cancer decreased by 95% in the young population, and the HCC-related mortality in elder generation is still unpromising (Chen et al., 2015 ; Kao, 2015). For HCC patients, most treatments focus on liver resection or transplantation; nevertheless, these methods cannot completely solve the high rates of recurrence (Forner et al., 2018).Thus, we need to find an effective way to solve this urgent issue.

Studies showed that in the whole genome, protein-coding genes are just the tip of the iceberg, more than 90% genes are the non-coding RNAs (ncRNAs) (Alexander et al., 2010; Esteller, 2011). Among them, long non-coding RNAs (lncRNAs) have attracted attention. LncRNAs are a kind of ncRNA with transcripts longer than 200nt in length and being involved in many elementary processes, including normal development, physiology and also disease (Mercer et al., 2009; Esteller, 2011). LncRNAs play profound roles at transcriptional and post-transcriptional levels of gene expression and regulate gene expression positively or negatively through various mechanisms (Chen, 2016). For example, lncRNAs can target chromatin regulators, as decoys to preclude the access of regulatory proteins to DNA. Besides, lncRNAs may also guide and recruit proteins to DNA. After transcribed by RNA polymerase II, some lncRNAs can function as signals and take an essential role in cis-regulation. As scaffolds, lncRNAs bring proteins into complexes and help specific protein complexes to localization (Wang and Chang, 2011; Rinn and Chang, 2012; Goff and Rinn, 2015; Quinn and Chang, 2016; Noh et al., 2018).

The aberrant expression of lncRNAs has also been testified in many cancers, especially in HCC. For instance, highly expressed lncRNA MCM3AP-AS1 through miR-194-5p/FOXA1 promotes the growth of HCC (Wang et al., 2019). By binding to heterogeneous nuclear ribonucleoprotein K, a novel lncRNA named p53-Stabilizing and Activating RNA promotes the interaction of hnRNPK and p53 and arrests p53-mediated pathway, ultimately inhibits cells proliferation and tumorigenicity (Qin et al., 2019). 

LncRNA Ak001796 (MIR4435-2 host gene), located on chromosome 2q13, was first presented to be involved in non-small cell lung cancer (Yang et al., 2015). It was reported that lncRNA Ak001796 as an oncogene highly expressed in lung cancer, esophageal squamous cell carcinoma and HCC (Yang *et al*., 2015; Han et al., 2019; Zong et al., 2019). Although AK001796 was reported to play roles in prognosis, the specific functions and downstream mechanisms in HCC are not fully understood.

Herein, we further explored the mechanism of AK001796 in HCC cells. Analyzing clinical data from The Cancer Genome Atlas (TCGA) and combining it with published research, we found that AK001796 was dramatically over-expressed in HCC compared with the adjacent tumor and closely correlated with poor prognosis. Further studies on mechanism revealed that AK001796 in cytoplasm upregulated GAB1 by regulation of miR-150 to promote HCC proliferation. Through analyzing the principle of AK001796/miR-150/GAB1 axis on the GAB1 downstream pathway, we concluded that AK001796 exerted its function of promoting proliferation in HCC cells by activating the phosphorylation of Akt and ERK1/2. Our findings might provide potential biomarkers for HCC progression and therapeutic targets in the future clinical treatment.

## Material and Methods

### Cell culture

Five cell lines were used in this study, including four kinds of HCC cell lines (HepG2, SMMC-7721, HUH7 and BEL-7402) and a normal liver epithelial cell line (L02). They were from the Cell Bank of Shanghai Institute of Biochemistry and Cell Biology. Cells were cultured in high Glucose DMEM (Gibco, USA) with 10% fetal bovine serum, 1% penicillin/streptomycin and cultured at 37 ℃ in the humidified atmosphere of 5% CO_2_. Cells were treated with 25 μM PD98059 (Beyotime, China, an ERK1/2 inhibitor) and 20 μM LY294002 (Beyotime, China, an AKT inhibitor) at 37 ℃ for 24 h in the corresponding studies.

### Plasmid construction and transfection

LncRNA and mRNA silencers (si-AK001796#1, si-AK001796#2, si-AK001796#3 and si-GAB1), miRNA mimics (miR-150 mimics), miRNA inhibitor (miR-150 inhibitor), negative control (NC), and inhibitor negative control (NC inhibitor) were designed by GenePharma (China). The predicted miRNA binding sites and mutations of binding sites were designed and inserted into the pcDNA3.1 vector (Promega, USA), which form the plasmid of AK001796-wt and AK001796-mut. Both of them were purchased from Genecreat (China). All of them were transfected with Lip2000 Transfection reagent (Invitrogen, USA) with optimal concentration, 20 μM for miRNA mimics, inhibitors, and siRNAs and 4 μg for plasmids. After transfection, the cells were cultured at 37 ℃ for subsequent studies.

### Total RNA isolation, reverse transcription reaction and quantitative PCR (qPCR)

Total RNA was extracted from cells using Trizol (TaKaRa, China) and organic reagent, confirming the quality and quantity by BioSpectrometer (Eppendorf, Germany). Later, RNA samples were reverse transcribed to cDNA with TransScriot Kit (Transgen, China). The reverse transcription of microRNA uses Bulge-Loop™ miRNA Primer kit (RIBOBIO, China), and lncRNA and mRNA use Oligo(dT) as primer. As for microRNA, 2 μg total RNA, 0.8 μl miR-150 RT primers (5 μM), 0.8 μl U6 RT primers (5 μM) were mixed with diethylpyrocarbonate (DEPC) water to 9 μl and incubated at 70 °C for 10 minutes then incubated at 4 °C for 2 minutes. The mixture was added with 1 μl RT/RI Enzyme Mix (TransGen, China) and 10 μl TS Reaction Mix (TransGen, China) incubated at 42 °C for 60 minutes, then at 85 °C for 5 minutes in PCR instrument (ExCell Bio G3, China). As for lncRNA and mRNA, 2 μg total RNA, 1 μl Oligo(dT) (TransGen, China), 1 μl RT/RI Enzyme Mix and 10 μl TS Reaction Mix were mixed with DEPC water to 20 μl, incubated at 42 °C for 60 minutes, then at 85 °C for 5 minutes. All conditions for the reverse transcription experiments were verified by multiple experiments of gradient of concentration followed by the instructions https://www.transgen.com/download/pdf/AE301_2022-12-25.pdf to ensure the efficiency of primers and the effect of reverse transcription. For the optimal concentration, using DEPC water dilutes cDNA 50 times as following qPCR template. qPCR was performed with 2×SYBR Green PCR Master Mix (Bimake, USA). GAPDH and U6 snRNA worked as endogenous controls for mRNA/lncRNA and miRNA. The total 20 μl reaction solution including 10 μl 2×SYBR Green, 2 μl cDNA template, 0.8 μl forward primer (10 μM), 0.8 μl reverse primer (10 μM) and 6.4 μl DEPC water. All primers were designed by (Sangon Biotech, China) and RiboBio (China). The data was analyzed by comparing the Ct and the result were made using 2^-ΔΔCT^ method. The primer sequences of reverse transcription and qPCR are shown in Table 1.

**Table 1 -:** The primer sequences of reverse transcription and qPCR.

Primers	Sequences (5’-3’)
GAPDH-forward	TCCTGGTATGACAACGAAT
GAPDH-reverse	GGTCTCTCTCTTCCTCTTG
U6 RT	AAAATATGGAACGCTTCACGAATTTG
U6-forward	CTCGCTTCGGCAGCACATATACT
U6-reverse	ACGCTTCACGAATTTGCGTGTC
MiR-150 RT	GTCGTATCCAGTGCAGGGTCCGAGGTATTCGCACTGGATACGACCACTGG
MiR-150-forward	TCGGCTTCTCCCAACCCTTGTAC
MiR-150-reverse	GTCGTATCCAGTGCAGGGTCCGAGGT
AK001796-forward	AATGACTGGATGGTCGCTGC
AK001796-reverse	GGTTGGAAAAGATGCTGGTGAC
GAB1-forward	GATGGTTCGTGTTACGCAGTG
GAB1-reverse	CGCTGTCTGCTACCAAGTAGAA

### Protein extraction and western blot

Total protein was extracted and separated from cells using RIPA (NCM, China) containing 1% ProtLytic Protease Inhibitor Cocktail (NCM, China) on the ice. The proteins were quantified with Pierce BCA Protein Assay Kit (Thermo, USA) using Enzyme standard instrument (BD, USA). Total protein was denatured with β-mercaptoethanol, and was separated by 10% SDS-PAGE electrophoresis. After electrophoresis, the gels that contained the target proteins were cropped according to the molecular marker running alongside the proteins. The proteins in the gels were then transferred onto PVDF membranes. After blocking with 5% defatted milk 2 hours at room temperature, they were incubated with antibody at 4 ℃ overnight, and detected by enhanced ECL reagents (NCM, China). The membranes show the target and control protein lines by using ImageQuant LAS4000 digital imaging system (GE, USA).The primary antibodies were as follows: Anti‐GAB1 (1:500; SAB4501060; Millipore, USA), anti‐ERK (1:4000; 67170-1-Ig; Proteintech, China), anti‐pERK (1:2000; ab201015; Abcam, UK), anti‐AKT (1:1000; WL0003b; Wanlei, China), anti‐pAKT (1:1000; WLP001a; Wanlei, China), anti‐bcl‐2 (1:1000; #15071; Cell Signaling Technology, USA), anti‐p21 (1:1000; #2947;Cell Signaling Technology, USA); anti‐p27 (1:1000, #3686;Cell Signaling Technology, USA), and anti‐GAPDH (1:4000; 60004-1-Ig; Proteintech, China). The secondary antibodies were as follows: HRP+ goat anti-rabbit IgG (1:5000; A0277; Beyotime, China) and HRP+ goat anti-mouse IgG (1:5000; A0286; Beyotime, China). The blots in the figures were from different sets of blots from separate electrophoresis with equal number of samples (40 ng protein) loaded, and only one target protein in each blot from each electrophoresis was detected. The density among the bands in each blot rather than in different blots was observed and compared. Some of the images of the blots lack of adequate length as the blots were cut prior to hybridization with antibodies and this issue doesn’t affect the interpretation of the blots at all. 

### Cell cycle and cell proliferation

After transfection of lncRNA silence and negative control 24 h, two kinds of hepatoma carcinoma cells were both stained with Cell Cycle Analysis Kit (Bioss, China) and then performed using flow cytometer (BD Accuri C6, US). Because double-stranded DNA combined with PI produces fluorescence, the results show the relative ratio of cells in G0/G1 phase, S phase, G2/M phase. For the colony formation assay, after transfection, cells cultured in 6-well plates at densities of 200-300 cells/well, and culture medium was replaced every 3-4 days. Approximately 15 days later, cells were stained with crystal violet. After transfection in 24-well plates for 24h, cells were passed into 96-well plates at a density of 800 cells/well density. At 24 h, 48 h, 72 h and 96 h after transfection, culture mediums were added 10 μl/well Enhanced Cell Counting Kit-8 (NCM, China) into each well. The absorbance was measured at 490nm using BioSpectrometer (Eppendorf, Germany).

### Luciferase reporter assay

The predicted miRNA binding sites and mutations of binding sites were synthesized and inserted into the pmirGLO vector, which formed the plasmid of wt-AK001796 and mut-AK001796. Both of them and the pmirGLO vector were purchased from GenePharma (China). The empty vector and two reporter plasmids were transfected with NC, miR-150, NC-in and miR-150 inhibitor into hepatoma carcinoma cells. At 48 h after transfection, the luciferase activity was determined by Dual-Luciferase Reporter Assay System (Promega, USA), according to manufacturer’s instructions. 

### Subcellular fraction location

Cells were fractioned and divided into nucleus and cytoplasm by using the Paris Kit (Life Technologies, USA) and Ultrasonic Cell Disruption System (Scientz, China). After reverse transcription and qPCR, the location of lncRNA was detected through analyzing the content of cDNA by comparing with U6, the nuclear control, and GAPDH, the cytoplasmic control. 

### Xenograft tumor formation assay

Purchased from HFK BIOSCIENCE (Beijing, China), four-week-old female SPF grade BALB/c nude mice (18-20 g) were housed under specific pathogen-free conditions and manipulated according to protocols approved by the Ethic Committee of Jilin University with approval number: (2017) research review (21). All procedures were carried out under animal house protocols to minimize potential confounders such as the order of treatments and measurements, animal numbers in each cage, and cage location, etc. to minimize the confounders. All animal experiments in our study complied with the ARRIVE guidelines and were carried out under the National Institutes of Health guide for the care and use of Laboratory animals (NIH Publications No. 8023, revised 1978). The mice were individually kept in cages with corn cob bedding on a ventilated rack on a 12 h/12 h light:/dark cycle at temperature of 24 °C and humidity of 55%. The mice had *ad libitum* access to rodent chow and water. The BALB/c nude mice are immunocompromised and ideal hosts for fast-growing tumor cells. They are hairless, so it is easier to evaluate xenograft tumor growth. There are no special requirements for BALB/c nude mouse strains and the gender of the nude mice has no-impact on the experimental outcome. 

Mice were randomly divided into two groups with 6 animals in each group including a control group and an experimental group. The animal experiment was repeated three times, so the total number of mice used was 36. We used as few animals as possible to meet statistical requirements. After feeding the mice for one week, HepG2 cells transfected with NC or si-AK001796#1 (1×10^6^/150μl sterile PBS) were injected into two sides of the armpit of the mice by double-blind method. After tumor implantation, the nude mice were fed for 14 days. Mice were excluded if they died or the planting of tumor cells were unsuccessful. No exclusion prior to study completion. At the conclusion of each experiment, the mice were euthanized by intraperitoneal barbital injection. The volume and weight of the tumors were measured using digital calipers and electronic balances. Volumes= π/6 (length × width^2^). Tumor samples from different groups were subjected to hematoxylin and eosin (HE) staining and immunohistochemistry for Ki-67 staining. During experiment, only the first author of this paper was aware of the group allocation at the different stages. The animal residues were then placed in a freezer and collected by a medical waste treating company. 

### Statistical analysis

Statistical analyses were performed with SPSS 19.0 and GraphPad Prism 7.0. Experiments of this study were performed in triplicate at least. T-tests were used to value the difference between two groups of data. The sequencing data and clinical information of liver cance patients were downloaded from TCGA data portal of TCGA-LIHC (https://portal.gdc.cancer.gov). Differential expression analysis was performed using DESeq2 package of R, version 3.6.1 (R Foundation, Vienna, Austria), with absolute of log2fold-change (│log2FC│) > 1 and p < 0.05 as threshold. Differences in the level of gene expression were analyzed using Student’s t-test. Univariate and multivariate Cox proportional hazards regression models were used to analyze potential factors associated with prognosis. Overall survival was estimated with the Kaplan-Meier method, and the log-rank test was employed to evaluate differences. One-way ANOVA analysis was used to determine the multi-sample analysis. Kaplan-Meier method was used to analyze the clinical characteristics of HCC patients. Data were presented as Mean ± SD from at least 3 independent experiments. P-value<0.05 was considered statistically significant. *P<0.05, **P<0.01, ***P<0.001.

## Results

### AK001796 is up-regulated in human HCC

**Figure 1 -:**
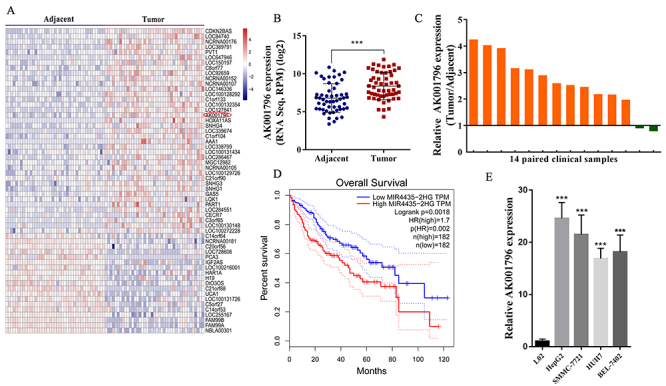
lncRNA AK001796 is up-regulated in human HCC. A. Hierarchical cluster heat map analysis (pheatmap packages of R studio) of differently expressed lncRNA in HCC tumor tissues and adjacent tissues generated from TCGA data base. Red in the map represented up-regulation and blue indicates down-regulation. The red circle indicated AK001796. B. Expression of AK001796 in TCGA cohorts. C. qPCR analysis of AK001796 expression in 14 paired of HCC and adjacent tissues from cDNA chip of Shanghai OUTDO Biotech (cDNA-HLivH030PG01). D. Overall survival was analyzed and compared on the basement of expression level of AK001796 in TCGA cohorts using the Kaplan-Meier method. E. AK001796 expression in HCC cell lines (HepG2, SMMC-7721, HUH7, BEL-7402) and normal hepatocyte (L02). (P=0.0002, P=0.0007, P=0.0002, P=0.0008, respectively.) Data were presented as Mean ± SD from at least 3 independent experiments. *** P < 0.001. TX: T stage unknown. NX:N stage unknown. MX: metastasis status unknown; NA: Not Available* Statistically significant results (in bold)

In this study, to find the aberrant expression of lncRNAs in HCC, we analyzed the clinical sequencing data of liver cancer tissue and their adjacent tissues downloaded from TCGA database. Among 58 aberrant expression of lncRNAs in HCC, 39 were up-regulated and 19 were down-regulated (Figure 1A). Among these, AK001796 initially drew our attention because of its high abundance in cancer tissues comparing to tumor adjacent (Figure 1B) (P<0.001). We then validated AK001796 expression in HCC tissues. In 14 paired clinical HCC tissue and adjacent tissue samples (from Shanghai OUTDO Biotech cDNA chip) by qPCR, AK001796 was obviously upregulated in 85.7% (12 of 14 paired samples) of the HCC tissue samples (Figure 1C). We further evaluated the relationship between the aberrant expressed lncRNAs and the overall survival of the HCC patients and found that high AK001796 expression was primarily correlated with poor survival of the HCC patients (P < 0.001) (Figure 1D). All the results above-mentioned made AK001796 a promising candidate for further study. To explore the clinical relevance of AK001796 in HCC, according to the median value of AK001796 expression, we divided the enrolled patients from TCGA datasets into two groups, respectively high expression of AK001796 expression and low expression of AK001796. As shown in Table 2, statistical analysis demonstrated that the expression level of AK001796 was correlated with tumor grade and pathological stage. We detected AK001796 expression in five cell lines (HepG2, SMMC-7721, HUH7, BEL-7402 and L02), and found that compared with L02, expression level of AK001796 was up-regulated in HCC cell lines (Figure 1E). 

**Table 2 -:** Correlations between the expression of AK001796 and clinical characteristics in HCC.

Characteristic	Low expression of AK001796	High expression of AK001796	P-value
n	187	187	
T stage, n (%)			< 0.001***
T1	113 (30.5%)	70 (18.9%)	
T2	30 (8.1%)	65 (17.5%)	
T3	38 (10.2%)	42 (11.3%)	
T4	4 (1.1%)	9 (2.4%)	
TX	3		
N stage, n (%)			1.000
N0	129 (50%)	125 (48.4%)	
N1	2 (0.8%)	2 (0.8%)	
NX	116		
M stage, n (%)			0.622
M0	134 (49.3%)	134 (49.3%)	
M1	1 (0.4%)	3 (1.1%)	
MX	102		
Pathological stage, n (%)			< 0.001***
Stage I	106 (30.3%)	67 (19.1%)	
Stage II	29 (8.3%)	58 (16.6%)	
Stage III	39 (11.1%)	46 (13.1%)	
Stage IV	1 (0.3%)	4 (1.1%)	
NA	24		
Tumor status, n (%)			0.095
Tumor free	109 (30.7%)	93 (26.2%)	
With tumor	68 (19.2%)	85 (23.9%)	
NA	19		
Gender, n (%)			0.658
Female	63 (16.8%)	58 (15.5%)	
Male	124 (33.2%)	129 (34.5%)	
Age, n (%)			0.643
<=60	86 (23.1%)	91 (24.4%)	
>60	101 (27.1%)	96 (25.5%)	

###  Silencing of AK001796 suppresses cell growth *in vitro*

**Figure 2 -:**
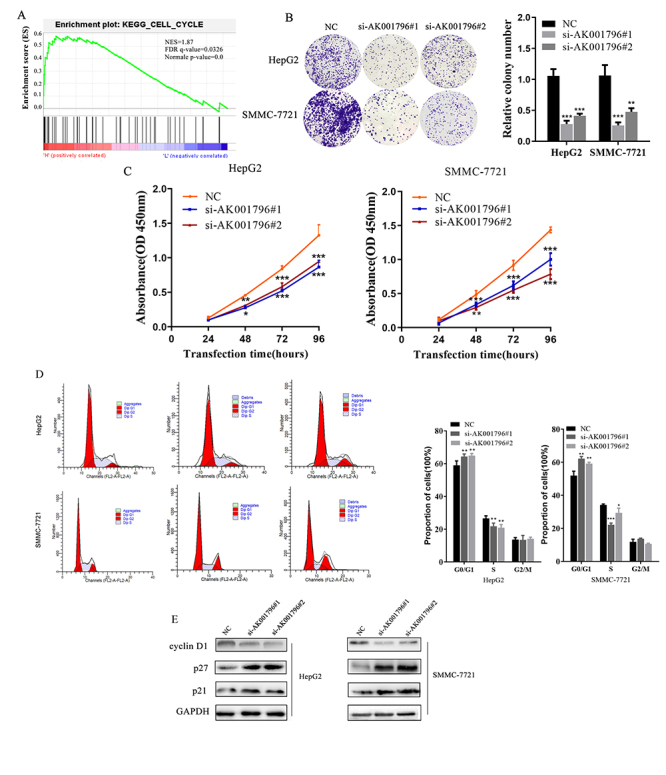
Depletion of AK001796 suppresses HCC cell growth and induces apoptosis *in vitro*. A. Results of gene set enrichment analysis (GSEA) were plotted to visualize the correlation between the expression of AK001796 and cell cycle related gene from GEO cohort. ‘H’ for high level of AK001796 and ‘L’ for low level of AK001796. B. HepG2 and SMMC-7721cells transfected with AK001796 siRNAs and negative control were seeded onto 6-well plates. The number of colony number was counted after 14 days. (P<0.0001, respectively.) C. HepG2 and SMMC-7721 cells transfected with AK001796 siRNAs and negative control were subjected to the CCK-8 assay 24 h intervals up to 96 h. (P<0.0001, respectively.) D. Flow cytometric cell cycle distribution assays to detect the proportion of HepG2 and SMMC-7721 in G0/G1, S, and G2/M phases after transfected with AK001796 siRNAs and negative control. (P<0.001, respectively.) E. Western blot analysis of CyclinD1, p27, and p21 expression in HepG2 and SMMC-7721 cells transfected with AK001796 siRNAs and negative control. The Western blot for cyclin D1, p21 and p27 were cropped from different gels. Data were presented as Mean ± SD from at least 3 independent experiments. *P < 0.05, **P < 0.01, *** P < 0.001.

To elucidate the role of AK001796 in HCC cells, we first analyzed the correlation between the AK001796 level and expression of many fundamental signal related genes in HCC with Gene Set Enrichment Analysis (GSEA). We found cell cyclin related genes were enriched in the group of AK001796 high level (Figure 2A). Later we designed three independent small interfering RNAs (siRNAs) (from GenePharma) to silence AK001796 expression. Depletion of AK001796 in HCC cells was validated by qPCR. (Figure S1A). si-AK001796#1 and si-AK001796#2 were validated to significantly suppress AK001796 expression, which were chosen to be used in further studies. Then CCK8 and colony formation assays indicated that silencing AK001796 suppressed the growth of HepG2 and SMMC-7721 cells (Figure 2B, C) (P<0.0001). Furthermore, flow cytometry cell cycle results showed that silencing AK001796 would induce cell arrest in G1 phrase and decrease the proportion of cells in S phrase (Figure 2D) (P<0.001). We further estimated some essential proteins related to cell cycle. These results manifested that silencing AK001796 decreased the expression level of CyclinD1 and increased the expression level of p21 and p27 in HCC cells (Figure 2E). Altogether, our results showed that the high level of AK001796 promoted cell growth in HCC cells.

### AK001796 promotes HCC proliferation *in vivo*

**Figure 3 -:**
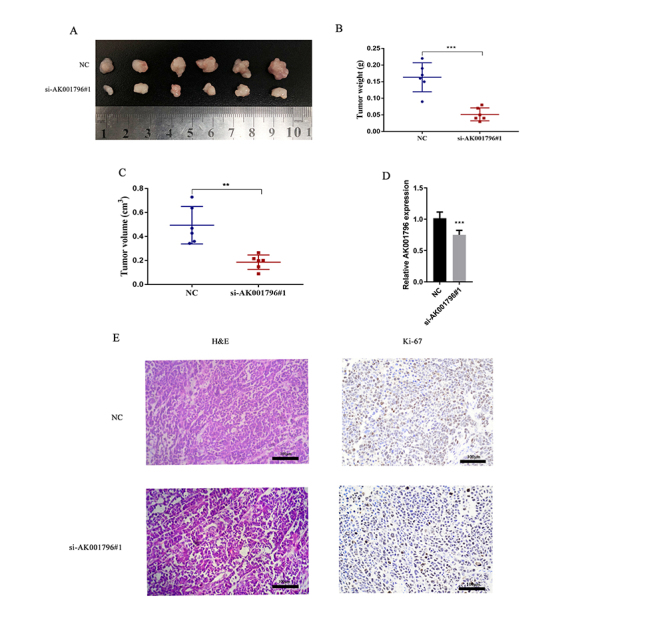
AK001796 promotes HCC proliferation *in vivo*. A. Representative images of tumors from the group of NC and si-AK001796#1. B-C. After transfecting negative control and si-AK001706#1 for 14 days, tumors weight and volume. (P=0.0002, P=0.0011, respectively.) D. AK001796 expression in tumors was detected by using qPCR. (P<0.0001) E. Representative imagines for hematoxylin and eosin (HE) staining and Ki-67 immunostaining of tumors samples from different groups are shown. Original magnification × 200. Scale bars = 100 μm. Data were presented as Mean ± SD from at least 3 independent experiments. **P < 0.01 and ***P < 0.001.

To evaluate the effect of AK001796 on tumor growth *in vivo*, we subcutaneously injected the indicated HCC cells, which were transfected with NC or si-AK001796#1, into 4-week nude mice to establish the xenograft tumor model. Fourteen days after the injection, compared with NC groups, tumors from the si-AK001796#1 group were obviously smaller and lighter (Figure 3A-C) (P=0.0002, P=0.0011), and had negative levels of AK001796 (Figure 3D) (P<0.0001). Besides, tumor tissues collected from si-AK001796#1 group exhibited less Ki67-positive rates (Figure 3E). These data indicated that AK001796 can influence HCC growth *in vivo*.

### AK001796 functions as a sponge for miR-150 in the cytoplasm

**Figure 4 -:**
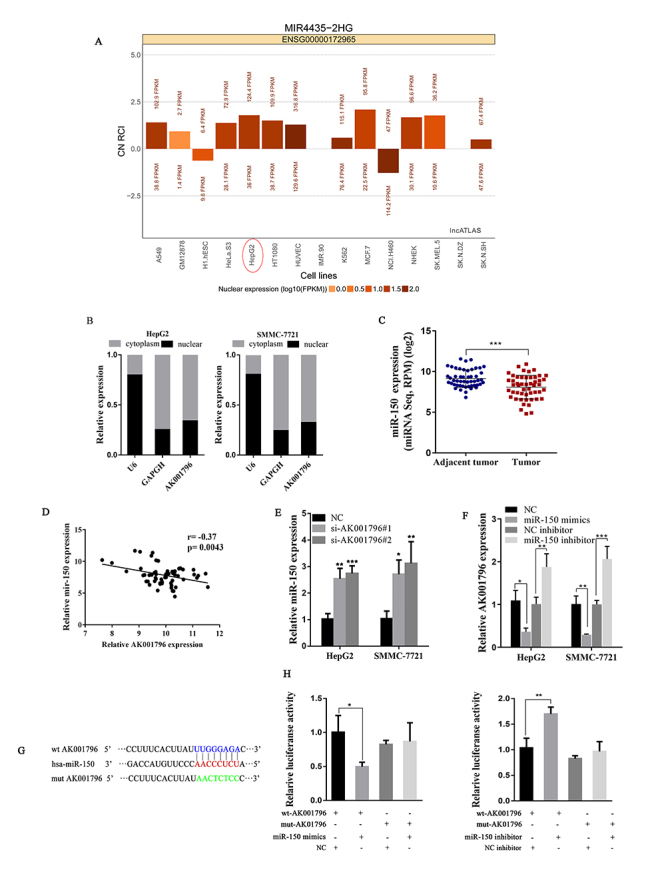
AK001796 functions as a sponge for miR-150 in the cytoplasm. A. The prediction of AK001796 subcellular localization by using LncATLAS, which was based on RNA sequencing. The red circle indicated AK001796 was mostly distributed in the cytoplasm of HCC. B. Relative expression levels of AK001796 in nuclear and cytosolic fractions of HepG2 and SMMC-7721 cells. (Nuclear controls: U6, Cytosolic controls: GAPDH). C. Expression of miR-150 in TCGA cohorts (P<0.001). D. The correlation between miR-150 and AK001796 expression analyzed in 60 paired colorectal cancer samples from TCGA (n = 60, r =−0.37, P = 0.0043). E. MiR-150 expression in HepG2 and SMMC-7721 cells transfected with AK001796 siRNAs and negative control. (P=0.0017, P=0.0009; P=0.0235, P=0.0088, respectively.). F. AK0001796 expression in HepG2 and SMMC-7721 cells transfected with miR-150 mimics, miR-150 inhibitor, negative control and inhibitor negative control (P=0.0129, P=0.0049; P=0.0052, P=0.0004, respectively.). G. Sequence of miR-150 and its predicted binding sites (red) in AK001796. Predicted miR-150 target sequence (blue) in AK001796 (wt-AK001796) and position of mutated nucleotides (green) in AK001796 (mut-AK001796). H. Luciferase reporter assay. A vector containing wild type AK001796 3′-UTR or mutant AK001796 3′-UTR was co-transfected into HCC cells together with miR-150 inhibitor or miR-150 mimics. Luciferase activity ratio was presented as firefly luciferase value/renilla luciferase value (P=0.0261, P=0.0081, respectively.). Data were presented as Mean ± SD from at least 3 independent experiments. * P < 0.05, ** P < 0.01, *** P < 0.001.

Emerging evidence suggested that there were several long non-coding RNAs regulating gene expression by participating in competitive endogenous RNAs regulatory networks in cytoplasm (Salmena et al., 2011). To further demonstrate whether the underlying molecular mechanism of AK001796 in HCC cells was involved in ceRNA theory, it was necessary to determine the subcellular localization of AK001796. Using LncATLAS (http://lncatlas.crg.eu/), a website of a complexed resource of lncRNA localization in human cells based on RNA-sequencing(Mas-Ponte et al., 2017), predicted the AK001796 localization in various cells. As Figure 4A shows, according the relative concentration index (RCI), we can conclude that in most cases, AK001796 was distributed in the cytoplasm especially in HepG2 cells. Furthermore, using Paris Kit for subcellular fractionation, we could see that compared with U6 and GAPDH by qPCR, AK001796 was mainly distributed in cytoplasm (Figure 4B). Then we predicted extensive microRNAs that might bind to AK001796 using LncBase v.2 of DIANA tools. We analyzed 28 microRNAs with a possible binding score greater than 0.95 (Table S1), and were surprised to find that only miR-150 was aberrantly expressed in HCC from Mircancer (http://mircancer.ecu.edu/). Intriguingly, it was reported that down-regulated miR-150 is related with proliferation in non-small cell lung cancer, colorectal cancer and follicular lymphoma (Lu et al., 2017; Chen et al., 2018; Musilova et al., 2018). Thus, we speculated that AK001796 might serve as the sponge of miR-150 in HCC. To test our hypothesis, we carried out further research. We analyzed the expression of miR-150, we found that it had lower abundance in cancer tissues compared to tumor adjacent of HCC patients downloaded from TCGA (Figure 4C) (P<0.001). Furthermore, a remarkable relationship between miR-150 level and AK001796 level was observed in 60 tumor samples from TCGA database (r = −0.37, P = 0.0043) (Figure 4D). We detected that the expression of miR-150 was up-regulated after transfecting si-AK001796#1 and si-AK001796#2 in HepG2 and SMMC-7721 cells (Figure 4E) (P<0.01). We designed miR-150 mimics and inhibitor to control miR-150 expression. Figure S1B showed that the effects of miR-150 mimics and inhibitors were validated by qPCR.

To determine whether miR-150 negatively regulates AK001796, we transfected miR-150 mimics or inhibitor into HepG2 and SMMC-7721 cells, and found that AK001796 expression had a significant suppression by miR-150 mimics, conversely, miR-150 inhibitor markedly enhanced AK001796 expression (Figure 4F) (P<0.01). Next, we determined the reciprocity between miR-150 and AK001796 in HCC by using dual luciferase reporter assays. Putative target sites of miR-150 in AK001796 and mutant type of AK001796 were shown in Figure 4G. Dual luciferase reporter assays showed a reduction of luciferase activity in HepG2 cells which co-transfected with miR-150 mimics and wt-AK001796 (pmirGLO contains the target sites) (P=0.0261). In contrast, we obtained the opposite results in HepG2 cells co-transfected with miR-150 inhibitor and mut-AK001796 (pmirGLO comprises the target sites) (P=0.0081) (Figure 4H). Taken together, AK001796 acted as a sponge for miR-150 in the HCC cytoplasm. 

### AK001796 promotes GAB1 expression through crosstalk with miR-150

**Figure 5 -:**
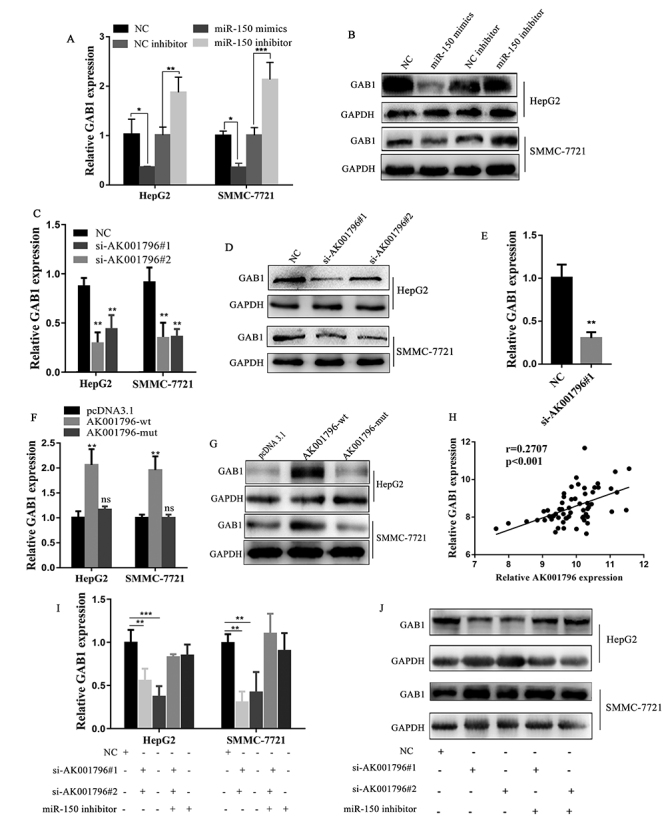
AK001796 promotes GAB1 expression through crosstalk with miR-150. A-B. Treatment with negative control, inhibitor negative control, miR-150 mimics and miR-150 inhibitor in HepG2 and SMMC-7721 cells, A was qPCR analysis of mRNA level of GAB1 and B was western blot analysis of protein level of GAB1. (P=0.0318, P=0.0077; P=0.0169, P=0.0005, respectively.). C-D. Treatment with negative control, AK001796 siRNAs in HepG2 and SMMC-7721 cells, C was qPCR analysis of mRNA level of GAB1 and D was western analysis of protein level of GAB1. (P=0.0013, P=0.0055; P=0.0033, P=0.0037, respectively.). E. GAB1 expression in xenograft tumors was detected by using qPCR (P=0.0019). F-G. Treatment with pcDNA3.1, AK001796-wt and AK001796-mut in HepG2 and SMMC-7721 cells, F was qPCR analysis of mRNA level of GAB1 and G was western analysis of protein level of GAB1. (P=0.0016, P=0.001, respectively.) H. The correlation between GAB1 and AK001796 expression analyzed in 60 paired colorectal cancer samples from TCGA (n = 60, r = 0.2707, P < 0.001). I-J. Co-transfection with negative control, AK001796 siRNAs and miR-150 inhibitor in HepG2 and SMMC-7721 cells, I was qPCR analysis of mRNA level of GAB1 and J was western blot analysis of protein level of GAB1. (P=0.0023, P=0.0002; P=0.0027, P=0.0092, respectively.) Data were presented as Mean ± SD from at least 3 independent experiments. * P < 0.05, ** P < 0.01, *** P < 0.001.

As the tumor suppressor gene, miR-150 could inhibit signaling molecules of proliferation via multiple ways (Xu et al., 2016; Cheng et al., 2017; Li et al., 2017). Among these, we focused on GAB1, a direct target of miR-150. More intriguingly, though our former results and prediction information from LncBase v.2, we found that AK001796 and GAB1 bound to the same target sites of miR-150 (Figure S1C) (P<0.001). Considering the former conclusion on relationship between AK001796 and miR-150, we speculated that AK001796 could sequester the interaction between miR-150 and GAB1, blocking the inhibition effect of miR-150 on GAB1 in HCC. To verify our hypothesis, we firstly respectively transfected miR-150 mimics and inhibitor into HepG2 and SMMC-7721 cells, and measured the expression level of GAB1 through qPCR and western blot. Results confirmed that overexpression of miR-150 suppressed GAB1 expression and inhibition of miR-150 upregulated GAB1 expression (Figure 5A-B) (P<0.05). After transfecting si-AK001796#1 and si-AK001796#2 in HepG2 and SMMC-7721 cells, the expression of GAB1 declined (Fig5C-D) (P<0.01). Next, we measured the expression of GAB1 from xenograft tumor tissues including si-AK001796#1 groups and NC groups, we found that GAB1 was reduced *in vivo* (Figure 5E) (P=0.0019). Besides, after inserting AK001796-wt (pcDNA3.1 contains the target sites), AK001796-mut (pcDNA3.1 contains mutant binding sites) and pcDNA3.1, qPCR and western blot analyses were performed to ascertain whether AK001796 acted as a sponge to sequester miR-150 to decrease the expression of its target, GAB1. These analyses revealed that wild type of AK001796 (including the target sites) but not the mutant increased GAB1 mRNA and protein levels in HCC cells. (Figure 5F-G) (P<0.01). Furthermore, a remarkable relationship between GAB1 level and miR-150 level was observed in 60 tumor samples from TCGA database (r = −0.2707, P < 0.001) (Figure 5H). Thus, we observed inhibition of miR-150 could rescue GAB1 both in mRNA and protein level attenuated by silencing AK001796 (Figure 5I-J) (P<0.01). These results strongly suggested that, AK001796 acted as a competing endogenous RNA to promote GAB1 expression by sponging miR-150 in HCC.

### AK001796 promotes proliferation by indirectly regulating GAB1 in HCC

**Figure 6 -:**
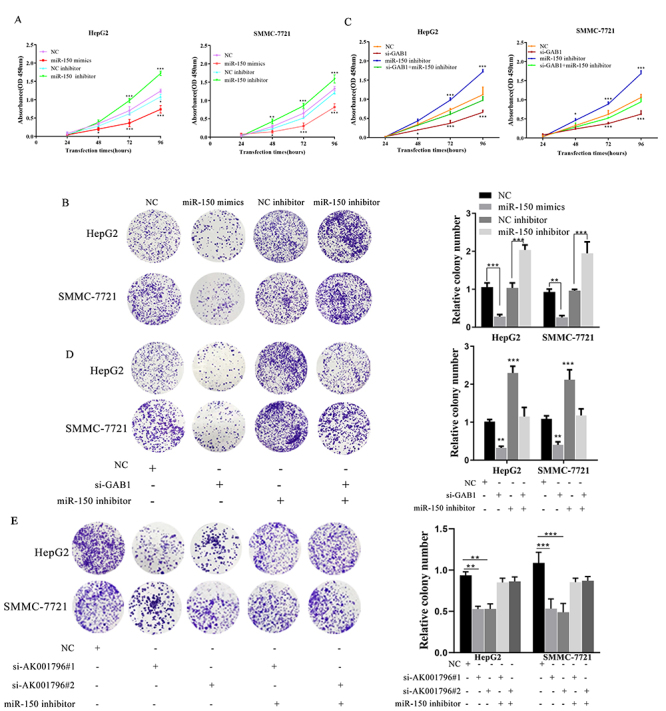
AK001796 promotes proliferation by indirectly regulating GAB1 in HCC. A-B. miR-150 mimics can restrain the HepG2 and SMMC-7721 proliferation, conversely miR-150 inhibitor facilitated the HepG2 and SMMC-7721 proliferation, as determined by CCK-8 proliferation assay (A) (P<0.0001), colony formation assay (B) (P=0.001, P<0.0001; P=0.0037, P=0.0003, respectively.). C-D. Reintroduction of miR-150 inhibitor into SMMC-7721 and HepG2 cells partially rescued the si-GAB1-mediated inhibition of cell proliferation, colony formation, as determined by CCK-8 proliferation assay (C) (P<0.0001), colony formation assay (D) (P=0.0025, P<0.0001; P=0.0045, P=0.0006, respectively.) E. Reintroduction of miR-150 inhibitor into SMMC-7721 and HepG2 cells partially rescued the si-AK001796-mediated inhibition of cell proliferation, colony formation, as determined colony formation assay (E) (P<0.01, P<0.0001, respectively). Data were presented as Mean ± SD from at least 3 independent experiments. * P < 0.05, ** P < 0.01, *** P < 0.001.

We designed si-GAB1, an independent small interfering RNA (siRNA) of GAB1 for gain/loss of function studies. As shown in Figure S1D-E, efficient changes in expression of GAB1 were tested. Subsequently, we performed a series of function research. In Figure 6A and 6C, CCK8 assay results indicated that miR-150 mimics can restrain the HCC growth; conversely miR-150 inhibitor facilitated the HCC growth. But silencing GAB1 could alleviate the growth acceleration of HCC cells induced by miR-150 inhibitor (P<0.0001). The colony assay displayed the same results as CCK8 (Figure 6B and 6D) (P<0.01). We could see that the inhibiting effects of miR-150 on HCC cells were counteracted by reintroducing GAB1. CCK-8 assay results showed that miR-150 inhibitor partially abolished the growth arrest of HepG2 and SMMC-7721 induced by AK001796 knockdown(S1F). The colony assays displayed the similar gain of function as CCK8 (Figure 6E) (P<0.01). These results confirmed that AK001796 regulated proliferation behaviors via miR‐150/GAB1 axis in HCC.

### AK001796 activates the ERK and Akt pathways through GAB1 to promote cell proliferation

**Figure 7 -:**
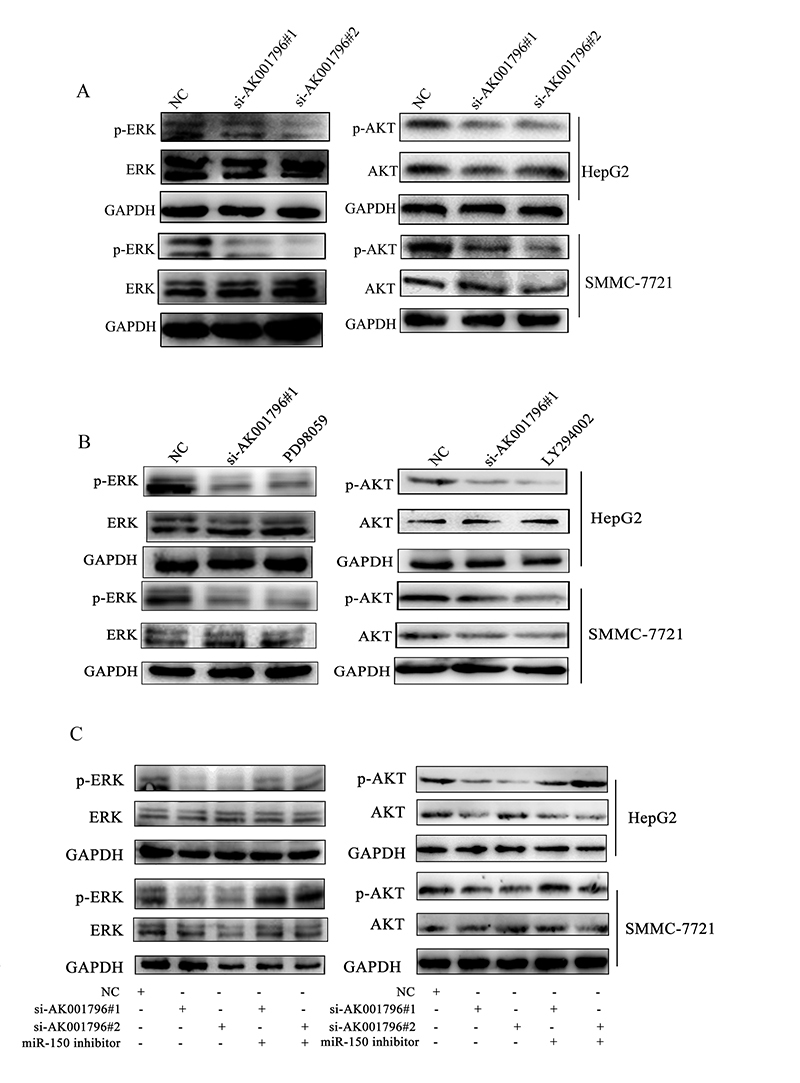
AK001796 activates the ERK and Akt pathways through GAB1 to promote cell proliferation. A. HepG2 and SMMC-7721 cells were transfected with negative control and AK001796 siRNAs. The levels of ERK1/2 and phospho-ERK1/2 (left) and the AKT and phospho-AKT (right) were evaluated by western blot analysis. B. HepG2 and SMMC-7721 cells were transfected with AK001796 siRNAs and negative control and were subjected to the PD98059 or LY294002. The levels of ERK1/2 and phospho-ERK1/2 (left) and the AKT and phospho-AKT (right) were evaluated by western blot analysis. C. MiR-150 inhibitor reintroduction into SMMC-7721 and HepG2 cells partially rescued si-AK001796-mediated inhibition of ERK (left) and AKT (right).

As is reported, Gab family is a highly conserved scaffold protein, which belongs to a kind of junction protein, and binds to growth factor receptor binding protein 2 (Gu and Neel, 2003; Nishida and Hirano, 2003). Some studies have demonstrated that GAB1 promoted cell proliferation, invasion and metastasis (Sang et al., 2013; Sang *et al*., 2015; Sun et al., 2016). GAB1 promotes the MAPK and AKT signaling pathway, important for regulating cell proliferation, migration and survival, etc. (Yamasaki et al., 2003; Chandrashekar et al., 2017; Xu et al., 2018a). As we mentioned that AK001796 could upregulate GAB1 expression through interacting with miR-150 and competing with GAB1, we speculated that AK001796 could activate ERK and Akt pathways through GAB1 to promote cell proliferation in HCC. Silencing the expression of AK001796 in HepG2 and SMMC-7721 cells, we found that phospho-ERK1/2 and phospho-AKT were both decreased (Figure 7A). Furthermore, we compared the inhibition effect of si-AK001796#1 in ERK and AKT signaling pathways with PD98059 (an inhibitor of ERK1/2 signaling pathway), and LY294002 (an inhibitor of PI3K/AKT signaling pathway) respectively. As displayed in Figure 7B, the inhibition effect on ERK signaling pathway between cells treated with si-AK001796#1 and PD98059 had no significant difference. Even though, the activation of ERK and AKT signaling pathways involving AK001796/miR-150/GAB1 axis still need to be clarified further. We transfected miR-150 inhibitor to explore the role of AK001796/miR-150 axis. As we expected, inhibiting miR-150 partially restored silencing AK001796-induced suppression of ERK and AKT signaling pathways (Figure 7C). These results suggested that AK001796 might function as an oncogene by indirectly increasing GAB1 expression and subsequent activation of downstream ERK and AKT signaling pathway in cytoplasm. In conclusion, AK001796/miR-150/GAB1 axis partly plays a momentous role in activating of ERK and AKT signaling pathway in HCC. 

## Discussion

HCC is one of the most diagnosed cancers, with a high fatality rate. Despite obvious advances in the treatment of HCC, only a few HCC patients are eligible for traditional treatment like potentially curative therapies and surgical resection (Bertuccio et al., 2017; Kim and Park, 2017; Bray et al., 2018). Non-coding RNAs have been widely reported to act as essential regulators in HCC. The crucial biological functions of non-coding RNAs include proliferation, invasion, metastasis, epithelial-mesenchymal transition (EMT) and autophagy in HCC (Klingenberg et al., 2017; Xu et al., 2018b). Yang et al firstly reported that lncRNA AK001796 acted as an oncogene in lung cancer carcinogenesis and participated in the anticancer effects of resveratrol (Yang *et al*., 2015). Besides, AK001796 was also newly discovered as an oncogene in HCC (Han et al., 2019). AK001796 has been found to regulate cell proliferation, cell cycle via modulating MDM2/p53 signaling in ESCC and increase the resistance of NSCLC cells to cisplatin by impacting cell apoptosis and cell proliferation (Liu et al., 2017; Liu *et al*., 2018). Up to now, there scarcely exist molecular mechanism studies of AK001796 in HCC.

In this study, we confirmed that AK001796 was highly expressed in tumor tissues and cells of HCC. The expression level of AK001796 also positively corelated with the tumor grade of HCC patients and negatively associated with the survival time of patients. We found that silencing AK001796 markedly restrained proliferation both in vitro and vivo by inducing cell arrest in G1 phase, decreasing the proportion of cells in S phase. These results indicated that AK001796 served as a prognostic factor.

In addition, we explored the mechanism of AK001796 in regulating the carcinogenesis of HCC. The effectiveness of lncRNA as ceRNA is influenced by its subcellular localization and numbers (Salmena et al., 2011). We predicted that miR-150 might bind to AK001796 by using LncBase v.2 of DIANA tools and verified this prediction via qPCR and dual luciferase report assays. We found that the expression of AK001796 was inversely associated with miR-150 levels. Thus, we concluded that AK001796 functions as a sponge for miR-150 in the HCC cytoplasm. But in the ceRNA theory, using MRE, the RNA communication language, mRNAs or other non-coding RNAs regulate their respective expression levels (Salmena *et al*., 2011), and ceRNAs just bind to microRNA, have few influence in its expression. Interestingly, we found that AK001796 and miR-150 expression could negatively regulate each other. At present, there exist few theories explaining this phenomenon, but there have been many studies on lncRNA function as microRNA sponge discovered this phenomenon (Zhang et al., 2013; Wang et al., 2016; Zhao et al., 2017; Han et al., 2019; Wang *et al*., 2019; Wang *et al*., 2020). Therefore, we will explore this phenomenon in future studies.

When phosphorylated tyrosine residues, GAB1 (Grb2-associated-binders family members) provide binding sites for effector proteins which playing principal roles in biological functions, including angiogenesis, invasion and metastasis (Bai et al., 2015; Wang et al., 2015; Wang *et al*., 2017). GAB1, as a kind of junction protein, which binds to growth factor receptor binding protein 2 and is associated with ERK and AKT pathway to regulate the growth migration and survival (Gu and Neel, 2003; Nishida and Hirano, 2003; Yamasaki et al., 2003; Lu et al., 2017; Xu et al., 2018a). Phosphatidylinositol 3-kinase (PI3K)-Akt and ERK-MAPK cascades positively contribute to glioma tumorigenesis and progression. Through reported studies and our results, we knew that GAB1 functioned as a ceRNA of miR-150 via binding to miR-150 directly and have the same target sites of miR-150 as AK001796. 

We performed a loss/gain of function approach to functionally characterize AK001796/miR-150/GAB1 axis in proliferation HCC. We verified silencing AK001796 can reduce the expression of GAB1 through ceRNA hypothesis and similarly reduce the level of phospho-ERK1/2 and phospho-AKT via western blot. 

Large numbers of researchers proposed the ceRNA can unify the transcriptome and form a large-scale regulatory RNA network by sharing MRE (Karreth and Pandolfi, 2013; Long et al., 2019). These networks could be used to gain insight into complex gene interactions and identify potential biomarkers to diagnose and treat HCC (Zhang et al., 2021). As an oncogene, AK001796 can target the miR-150 and increase GAB1 expression, which induces subsequent downstream ERK and AKT activation in HCC. The downstream genes of AK001796 formed a regulatory axis to regulate the proliferation of HCC (Figure S1G). We provided evidence indicating that AK001796 acted as a sponge for miR-150 and released its target of GAB1, increasing ERK and AKT activation in HCC. In this essay, we focused on the role of AK001796 and AK001796/miR-150/GAB1 axis in the cell proliferation of HCC., and confirm AK001796 can be used a potential biomarker and AK001796/miR-150/GAB1 axis may be a therapeutic way against HCC. The diverse action modes of lncRNA are related to its subcellular localization, we concluded that in most cases, AK001796 was distributed in the cytoplasm especially in HepG2 cells. In the future, we will verify whether AK001796 in the nucleus can be related to our AK001796/miR-150/GAB1 through other pathways. 

## Conclusions

Our study provided a novel molecular mechanism for AK001796 promoting HCC proliferation. Our study indicated that AK001796 was one of the GAB1 competing endogenous RNAs, could partly increase the expression of phospho-ERK1/2 and phospho-AKT in HCC. Our results suggested a strategy for targeting AK001796 as a potential biomarker and AK001796/miR-150/GAB1 might be a therapeutic agent in the treatment of HCC.

## Supplementary material

The following online material is available for this article:

Table S1 - Database of microRNAs that might bind to AK001796.

Figure S1 - Expression data and bioinformatics prediction.
