# Supplementary material for: LncRNA AK001796 promotes cell proliferation via acting as a ceRNA of miR-150 in hepatocellular carcinoma
Source: Genet Mol Biol. 2023 Jun 2;46(2):e20220277. doi: 10.1590/1678-4685-GMB-2022-0277 (PMC10240574; doi:10.1590/1678-4685-GMB-2022-0277)
Supplement: Table S1 - [file 1415-4757-GMB-46-2-e20220277-s1.pdf]

Genetics and Molecular Biology
https://doi.org/10.1590/1678-4685-GMB-2022-0277

Supplementary Material to "LncRNA AK001796 promotes cell proliferation via acting as a ceRNA of miR-150 in hepatocellular carcinoma"

Table S1- Database of microRNAs that might bind to AK001796.

#Transcript_ID	Gene_ID(Gene_Name)	Mirna_Name (miRBase_version)	score
>ENST00000409569	ENSG00000172965(MIR 4435-1HG)	hsa-miR-506-5p	0.999
2:310-337	8mer	0.117	
>ENST00000409569	ENSG00000172965(MIR 4435-1HG)	hsa-miR-4270	0.998
2:313-330	8mer	0.113	
>ENST00000603827	ENSG00000172965(MIR 4435-1HG)	hsa-miR-6512-3p	0.998
2:1272-1298	8mer	0.091	
2:785-799	9mer	0.015	
2:1207-1225	6mer	0.004	
>ENST00000609220	ENSG00000172965(MIR 4435-1HG)	hsa-miR-2052	0.997
2:22-34	8mer	0.077	
2:128-140	7mer	0.029	
>ENST00000603827	ENSG00000172965(MIR	hsa-miR-6720-5p	0.995

#Transcript_ID	Gene_ID(Gene_Name)	Mirna_Name (miRBase_version)	score
	4435-1HG)		
2:1272-1286	8mer	0.078	
2:785-811	9mer	0.016	
2:1207-1231	6mer	0.004	
>ENST00000603827	ENSG00000172965(MIR 4435-1HG)	hsa-miR-6733-5p	0.992
2:380-398	8mer	0.068	
2:59-77	7mer	0.014	
2:1336-1352	6mer	0.007	
>ENST00000603827	ENSG00000172965(MIR 4435-1HG)	hsa-miR-215-3p	0.991
2:245-262	8mer	0.088	
>ENST00000419736	ENSG00000172965(MIR 4435-1HG)	hsa-miR-215-3p	0.991
2:255-272	8mer	0.088	
>ENST00000442293	ENSG00000172965(MIR 4435-1HG)	hsa-miR-215-3p	0.991
2:244-261	8mer	0.087	
>ENST00000308604	ENSG00000172965(MIR 4435-1HG)	hsa-miR-215-3p	0.99
2:248-265	8mer	0.086	
>ENST00000603310	ENSG00000172965(MIR 4435-1HG)	hsa-miR-215-3p	0.985
2:245-262	8mer	0.079	

#Transcript_ID	Gene_ID(Gene_Name)	Mirna_Name (miRBase_version)	score
>ENST00000409569	ENSG00000172965(MIR 4435-1HG)	hsa-miR-215-3p	0.982
2:258-275	8mer	0.076	
>ENST00000604981	ENSG00000172965(MIR 4435-1HG)	hsa-miR-215-3p	0.982
2:245-262	8mer	0.076	
>ENST00000441075	ENSG00000172965(MIR 4435-1HG)	hsa-miR-215-3p	0.981
2:124-141	8mer	0.075	
>ENST00000432818	ENSG00000172965(MIR 4435-1HG)	hsa-miR-6722-5p	0.978
2:540-560	8mer	0.073	
>ENST00000609220	ENSG00000172965(MIR 4435-1HG)	hsa-miR-548n	0.974
2:4-23	8mer	0.049	
2:432-448	7mer	0.013	
2:683-709	6mer	0.008	
>ENST00000603827	ENSG00000172965(MIR 4435-1HG)	hsa-miR-6739-5p	0.974
2:380-405	8mer	0.049	
2:59-78	7mer	0.014	
2:1336-1340	6mer	0.007	
>ENST00000431385	ENSG00000172965(MIR 4435-1HG)	hsa-miR-4659b-3p	0.967
2:577-592	8mer	0.03	

#Transcript_ID	Gene_ID(Gene_Name)	Mirna_Name (miRBase_version)	score
2:197-215	8mer	0.019	
2:506-524	7mer	0.012	
2:559-575	6mer	0.005	
>ENST00000439362	ENSG00000172965(MIR 4435-1HG)	hsa-miR-188-3p	0.966
2:6-26	8mer	0.066	
>ENST00000442293	ENSG00000172965(MIR 4435-1HG)	hsa-miR-6885-3p	0.966
2:4-9	7mer	0.066	
>ENST00000431385	ENSG00000172965(MIR 4435-1HG)	hsa-miR-4659a-3p	0.966
2:577-595	8mer	0.03	
2:197-211	8mer	0.019	
2:506-524	7mer	0.012	
2:559-573	6mer	0.005	
>ENST00000605570	ENSG00000172965(MIR 4435-1HG)	hsa-miR-4732-5p	0.966
2:71-96	9mer	0.055	
2:934-961	6mer	0.005	
2:1031-1054	6mer	0.005	
>ENST00000439362	ENSG00000172965(MIR 4435-1HG)	hsa-miR-3138	0.965
2:386-413	8mer	0.063	
2:236-259	6mer	0.002	

#Transcript_ID	Gene_ID(Gene_Name)	Mirna_Name (miRBase_version)	score
>ENST00000603827	ENSG00000172965(MIR 4435-1HG)	hsa-miR-150-5p	0.964
2:288-308	7mer	0.033	
2:1046-1059	8mer	0.028	
2:826-841	6mer	0.003	
>ENST00000605570	ENSG00000172965(MIR 4435-1HG)	hsa-miR-629-5p	0.963
2:157-174	8mer	0.064	
>ENST00000371162	ENSG00000172965(MIR 4435-1HG)	hsa-miR-6778-3p	0.96
2:1936-1947	8mer	0.024	
2:94-115	8mer	0.018	
2:129-152	7mer	0.008	
2:565-588	7mer	0.007	
2:400-418	6mer	0.003	
2:122-137	6mer	0.003	
>ENST00000609220	ENSG00000172965(MIR 4435-1HG)	hsa-miR-7152-5p	0.957
2:123-142	8mer	0.058	
2:625-647	6mer	0.003	
>ENST00000603827	ENSG00000172965(MIR 4435-1HG)	hsa-miR-873-3p	0.955
2:519-546	8mer	0.049	
2:41-62	7mer	0.007	

#Transcript_ID	Gene_ID(Gene_Name)	Mirna_Name (miRBase_version)	score
2:1222-1241	6mer	0.004	
2:805-817	6mer	0.001	
>ENST00000308604	ENSG00000172965(MIR 4435-1HG)	hsa-miR-6764-5p	0.955
2:448-463	7mer	0.025	
2:475-490	7mer	0.016	
2:160-179	7mer	0.01	
2:293-313	6mer	0.009	
>ENST00000603827	ENSG00000172965(MIR 4435-1HG)	hsa-miR-6885-3p	0.955
2:5-10	7mer	0.061	
>ENST00000609902	ENSG00000172965(MIR 4435-1HG)	hsa-miR-627-3p	0.954
2:348-372	9mer	0.042	
2:431-448	8mer	0.018	
>ENST00000308604	ENSG00000172965(MIR 4435-1HG)	hsa-miR-6885-3p	0.953
2:8-13	7mer	0.056	
2:443-469	6mer	0.004	
>ENST00000371162	ENSG00000172965(MIR 4435-1HG)	hsa-miR-7151-3p	0.953
2:148-174	8mer	0.023	
2:585-598	7mer	0.013	
2:283-308	7mer	0.013	

#Transcript_ID	Gene_ID(Gene_Name)	Mirna_Name (miRBase_version)	score
2:452-478	8mer	0.01	
2:1012-1026	6mer	0.002	
>ENST00000603310	ENSG00000172965(MIR 4435-1HG)	hsa-miR-6885-3p	0.953
2:5-10	7mer	0.06	
>ENST00000439362	ENSG00000172965(MIR 4435-1HG)	hsa-miR-4732-5p	0.953
2:310-335	9mer	0.057	
2:973-998	6mer	0.003	
>ENST00000439494	ENSG00000172965(MIR 4435-1HG)	hsa-miR-6512-3p	0.952
2:446-458	8mer	0.032	
2:635-658	8mer	0.028	
>ENST00000419736	ENSG00000172965(MIR 4435-1HG)	hsa-miR-6885-3p	0.951
2:15-20	7mer	0.055	
2:450-457	6mer	0.005	
>ENST00000439494	ENSG00000172965(MIR 4435-1HG)	hsa-miR-6720-5p	0.95
