# Supplementary material for: LncRNA AK001796 promotes cell proliferation via acting as a ceRNA of miR-150 in hepatocellular carcinoma
Source: Genet Mol Biol. 2023 Jun 2;46(2):e20220277. doi: 10.1590/1678-4685-GMB-2022-0277 (PMC10240574; doi:10.1590/1678-4685-GMB-2022-0277)
Supplement: Figure S1 - [file 1415-4757-GMB-46-2-e20220277-s2.pdf]

*Genetics and Molecular Biology*
https://doi.org/10.1590/1678-4685-GMB-2022-0277

# Supplementary Material to "LncRNA AK001796 promotes cell proliferation via acting as a ceRNA of miR-150 in hepatocellular carcinoma"

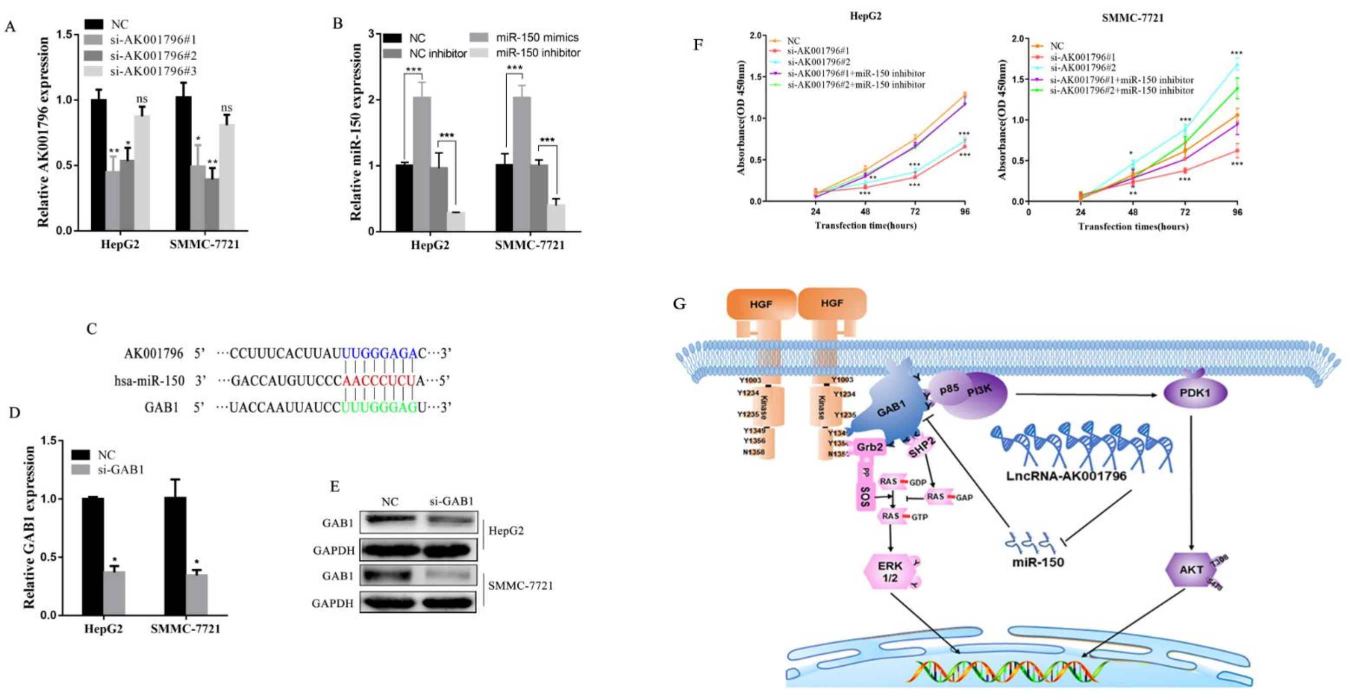

**Figure S1 -** Expression data and bioinformatics prediction. A. AK001796 expression was evaluated by qPCR in HepG2 and SMMC-7721 cells transfected with three AK001796 siRNAs. (P=0.0003, P=0.0009; P=0.0014, P=0.0004, respectively.) B. MiR-150 expression was evaluated by qPCR in HepG2 and SMMC-7721 cells transfected with negative control, inhibitor negative control, miR-150 mimics and miR-150 inhibitor. (P=0.0003, P=0.0048; P=0.0001, P=0.0037, respectively.) C. Bioinformatics predicted AK001796(blue) and GAB1(green) binding sites in miR-150 (red). D-E. GAB1 expression was evaluated by qPCR (D) (P<0.0001, n=3; P=0.0023, n=3.) and Western blot (E). F. Reintroduction of miR-150 inhibitor into SMMC-7721 and HepG2 cells partially rescued the si-AK001796-mediated inhibition of cell proliferation, colony formation, as determined by CCK8 assay. G. Schematic of the underlying mechanism of AK001796 in HCC using elements of ScienceSlides (2005) and Powerpoint software (2010). AK001796 functions as a ceRNA to sponge miR-150 and upregulated the expression of GAB1. AK001796/miR-150/GAB1 axis could promote proliferation of HCC by activating downstream signal pathway including ERK and Akt. Data were presented as Mean ± SD from at least 3 independent experiments. * P < 0.05, ** P < 0.01, *** P < 0.001.
